# Design and Construction of a Whole Cell Bacterial 4-Hydroxyphenylacetic Acid and 2-Phenylacetic Acid Bioassay

**DOI:** 10.3389/fbioe.2015.00088

**Published:** 2015-06-16

**Authors:** Seppe Dierckx, Sandra Van Puyvelde, Lyn Venken, Wolfgang Eberle, Jos Vanderleyden

**Affiliations:** ^1^Centre of Microbial and Plant Genetics, KU Leuven, Leuven, Belgium; ^2^Diagnostic Bacteriology Unit, Department of Biomedical Sciences, Institute of Tropical Medicine, Antwerp, Belgium; ^3^Imec, Leuven, Belgium

**Keywords:** auxin, bacterial biosensor, pyocyanin, GFP, 4-hydroxyphenylacetic acid, 2-phenylacetic acid

## Abstract

**Introduction:**

Auxins are hormones that regulate plant growth and development. To accurately quantify the low levels of auxins present in plant and soil samples, sensitive detection methods are needed. In this study, the design and construction of two different whole cell auxin bioassays is illustrated. Both use the auxin responsive element HpaA as an input module but differ in output module. The first bioassay incorporates the *gfp* gene to produce a fluorescent bioassay. Whereas the second one utilizes the genes *phzM* and *phzS* to produce a pyocyanin producing bioassay whose product can be measured electrochemically.

**Results:**

The fluorescent bioassay is able to detect 4-hydroxyphenylacetic acid (4-HPA) and 2-phenylacetic acid (PAA) concentrations from 60 μM to 3 mM in a dose-responsive manner. The pyocyanin producing bioassay can detect 4-HPA concentrations from 1.9 to 15.625 μM and PAA concentrations from 15.625 to 125 μM, both in a dose-responsive manner.

**Conclusion:**

A fluorescent whole cell auxin bioassay and an electrochemical whole cell auxin bioassay were constructed and tested. Both are able to detect 4-HPA and PAA at concentrations that are environmentally relevant to plant growth.

## 1 Introduction

Auxins are phytohormones that play an important role in every aspect of plant growth and development (Santner et al., 2009; Zhao, 2010). Because auxins are present at low concentrations in soil and plants, it is difficult to accurately determine environmental concentrations (Yin et al., 2010). To study the effects of auxins on plant growth, an efficient, sensitive method is therefore needed to adequately quantify concentrations of auxins in soil and plant samples. Environmental auxin concentrations are currently measured using high-performance liquid chromatography (HPLC) (Lu et al., 2010; Liang et al., 2012). HPLC experiments, however, require laborious preparations and extensive training (Shelver and Smith, 2003; Liang et al., 2012). Biosensors offer a low effort and low complexity alternative for the detection of auxins. They consist of a biological responsive element, which binds an analyte and a physical detector that in turn transduces the binding to a measurable signal. The most widely used biological responsive elements in biosensors are enzymes (Yagi, 2007). Enzymes are highly selective, but need to be purified before they can be used (Freguia et al., 2012), which is often expensive and labor-intensive (D’Souza, 2001; Yagi, 2007; Freguia et al., 2012). In recent years, the whole cell bacterial bioassay, a biosensor in the form of a complete bacterial cell, has emerged as an alternative (D’Souza, 2001). Purification is no longer required and everything needed for the function of the sensor is already present in a self-replicating system. Although an initial effort is required for the construction of the whole cell bacterial bioassay, their application is relatively inexpensive and easy (Hansen and Sørensen, 2001). Several different whole cell bacterial bioassays have already been constructed for use in soil environments to monitor essential resources for plant growth. These include biosensors for iron (Joyner and Lindow, 2000), sucrose, tryptophan (Jaeger et al., 1999), arsenate/arsenite (Stocker et al., 2003), and phosphate (Kragelund et al., 1997). However, currently no biosensor exists for the detection of environmental auxin concentrations.

**Figure 1:**
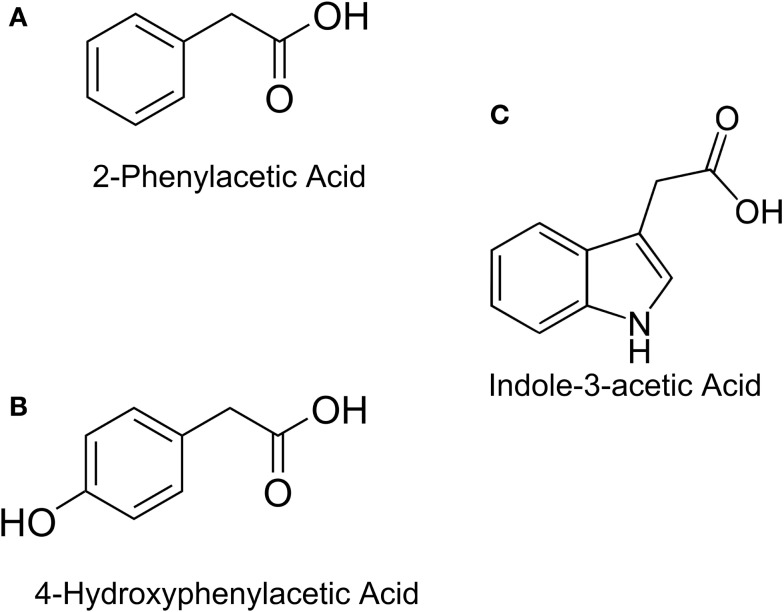
**Chemical structures of: (A) Indole-3-acetic Acid (IAA), (B) 2-Phenylacetic Acid (PAA), (C) 4-Hydroxyphenylacetic acid (4-HPA)**.

**Figure 2:**
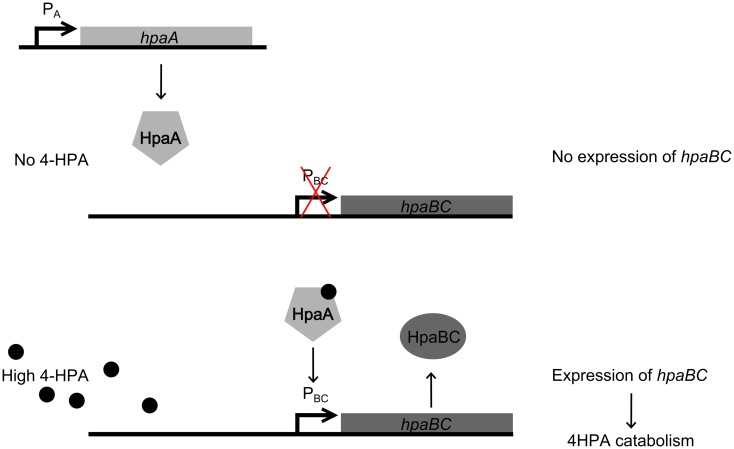
**Regulation of operon *hpaBC* by HpaA**. (top) In the absence of 4-Hydroxyphenylacetic acid (4-HPA), HpaA does not bind to promoter P*_BC_*, therefore HpaBC is not expressed. (bottom) When 4-HPA is present and binds to HpaA, HpaA in turn binds to P*_BC_* and promotes the expression of *hpaBC*.

In this study, the design and construction of a whole cell bacterial auxin bioassay for the detection of auxins, 2-phenylacetic acid (PAA) (Figure 1A) and PAA derivative 4-hydroxyphenylacetic acid (4-HPA) (Figure 1B), combined with two different output systems is illustrated. First, an auxin responsive element to function as input module for the bioassay was chosen. PAA and 4-HPA catabolic pathways in *Escherichia coli*, of which several have already been identified (Ferrández et al., 2000; Galán et al., 2003), provided such a responsive element. Specifically, transcription factor HpaA of operon *hpaBC* (Prieto and García, 1997) was selected. HpaA is present in *E. coli* W (ATCC 11105), a bacterial strain that has the innate ability to metabolize aromatic compounds like 4-HPA, contrary to *E. coli* K-12 strains that do not possess this ability (Prieto et al., 1996a). Prieto et al. observed that *hpaBC* codes for a two component aromatic hydroxylase responsible for the first step of the 4-HPA catabolism (Prieto et al., 1996a,b; Prieto and García, 1997). Its expression is regulated by transcription factor HpaA, encoded by gene *hpaA*, expressed constitutively by promoter P*_A_*. The mechanism is schematically shown in Figure 2. Once HpaA is bound to 4-HPA, a conformational change occurs, allowing the HpaA–4-HPA complex to bind to promoter P*_BC_*. After binding the promoter, the expression of HpaBC, downstream of promoter P*_BC_*, is induced. HpaBC in turn will break down 4-HPA (Prieto et al., 1996b; Prieto and García, 1997). The ability of HpaA to induce expression of genes downstream of promoter P*_BC_* after binding 4-HPA allows it to be used as biological responsive element for a 4-HPA bioassay. PAA has a similar structure to 4-HPA and can also bind to HpaA (Prieto and García, 1997), which can therefore be used to detect both 4-HPA and PAA. Finally, HpaA was also used in combination with indole-3-acetic acid (IAA) (Figure 1C), another auxin with a different structure from 4-HPA and PAA, to verify the selectivity of the bioassay.

In addition to an input module, a functional whole cell bioassay also requires an appropriate output module. Here, two different reporter systems were coupled to the HpaA input module. The first one is the *gfp* gene, whose gene product, green fluorescent protein (GFP), can be measured optically. The second reporter system is based on the redox-active molecule pyocyanin that can be detected electrochemically using cyclic voltammetry (CV) (Vukomanovic et al., 1996; Bukelman et al., 2009). Many biologically relevant molecules are oxidized when a voltage is applied but most oxidize at positive voltages opposed to a Ag/AgCl electrode. Pyocyanin on the other hand oxidizes at a negative voltage (−247 mV). This means that, at these voltages only phenazines will be detected (Webster et al., 2014). Pyocyanin is therefore an interesting candidate for use as a bacterial output product because there is only a small chance of a false positive signal with other products. Pyocyanin is a redox-active blue-pigment phenazine and one of the virulence factors of *Pseudomonas aeruginosa* (Mavrodi et al., 2001). When a voltage of −247 mV is applied, pyocyanin will oxidize, producing a measurable current proportional to the concentration of pyocyanin present. The biosynthesis of pyocyanin from chorismic acid in *P. aeruginosa* is complex and involves two homologous operons (*phzA1B1C1D1E1F1G1* and *phzA2B2C2-D2E2F2G2*), to produce phenazine-1-carboxylic acid (PCA) from chorismic acid, and two additional genes (*phzM* and *phzS*) whose gene products convert PCA to 5-methylphenazine-1-carboxylic acid (5-MCA) and 5-MCA to pyocyanin, respectively (Mavrodi et al., 2001).

This work describes the design and construction of a whole cell bacterial auxin bioassay which uses auxin responsive element HpaA for the detection of 4-HPA and PAA, in combination with both a fluorescent reporter system (GFP) and an electrochemical reporter system (pyocyanin).

## 2 Results

### 2.1 Whole cell auxin bioassay using GFP as output module

**Figure 3:**
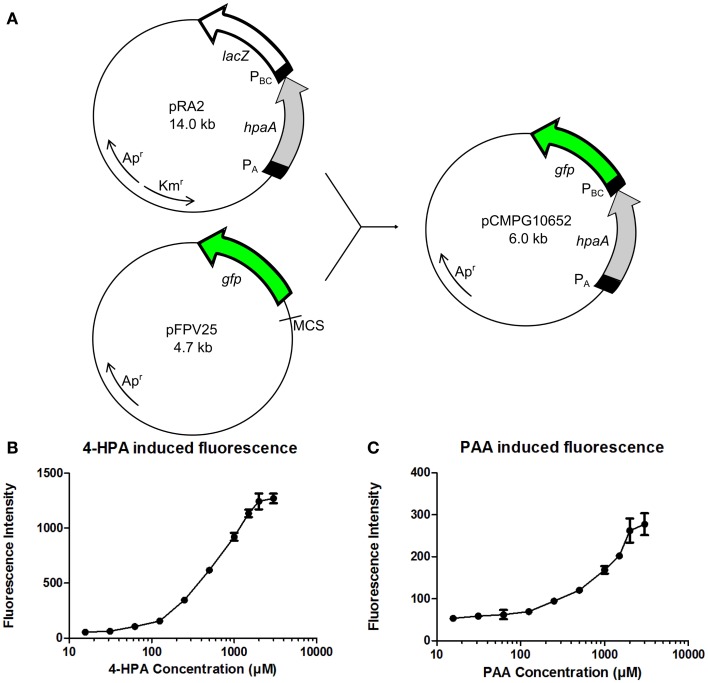
**(A)** Construction of plasmid pCMPG10652. Constitutive promoter P*_A_*, gene *hpaA*, and 4-Hydroxyphenylacetic acid (4-HPA) responsive promoter P*_BC_* were copied from plasmid pRA_2_ (Prieto and García, 1997) and placed in plasmid pFPV25 (Valdivia and Falkow, 1996), upstream of gene *gfp*. This yields GFP production controlled by 4-HPA and 2-Phenylacetic Acid (PAA) concentrations, detected by HpaA. **(B)** Fluorescence measurements of GFP production of strain pCMPG10652/*E. coli* TOP10 in response to a range of 4-HPA concentrations. **(C)** Fluorescence measurements of GFP production of strain pCMPG10652/*E. coli* TOP10 in response to a range of PAA concentrations. Auxin concentrations used in fluorescence measurements: 3 mM, 2 mM, 1.5 mM, 1 mM, 500 μM, 250 μM, 125 μM, 62 μM, 31.25 μM, 15.625 μM, and 0M. *n* = 8 per measured concentration.

The GFP reporter system is relatively simple, consisting only of the *gfp* gene which is present in plasmid pFPV25 preceded by a multiple cloning site (MCS) (Valdivia and Falkow, 1996) (Figure 3A). pFPV25 is therefore often used to study the activity of unknown promoters (Bijlsma and Groisman, 2005). For the construction of the fluorescent whole cell bacterial bioassay, the DNA sequence of plasmid pRA_2_ (Prieto and García, 1997) containing the constitutive P*_A_* promoter, the *hpaA* gene, and the 4-HPA responsive promoter P*_BC_* was amplified and placed upstream of the *gfp* gene in plasmid pFPV25. This resulted in plasmid pCMPG10652.

The expression of GFP in *E. coli* TOP10 bacteria carrying pCMPG10652, in response to different concentrations of 4-HPA, PAA, and IAA, was determined using fluorescence measurements. In order to determine the influence of the bacterial growth phase on GFP production in response to auxin concentrations, pCMPG1062/TOP10 bacteria were exposed to different auxin concentrations during either lag phase, exponential phase, or stationary phase. Fluorescence measurements of these cultures were performed every 2 h for 24 h (Figures S1–S9 in Supplementary Material). Results indicate a dose-response relationship when bacteria were exposed to auxins during the exponential phase, in response to both 4-HPA and PAA. This dose-response relationship is apparent in the presence of 4-HPA concentrations from 60 μM to 3 mM (Figure 3B) after an incubation period of 10 h. At a concentration of 3 mM, a plateau develops. A dose-response relationship is observed in the presence of PAA concentrations between 60 μM and 3 mM (Figure 3C). Of note is that PAA elicits over four times less GFP production than 4-HPA in the presence of similar concentrations of auxin. There was no visible production of GFP in response to IAA (Figures S7–S9 in Supplementary Material).

### 2.2 Whole cell auxin bioassay using pyocyanin as output module

**Figure 4:**
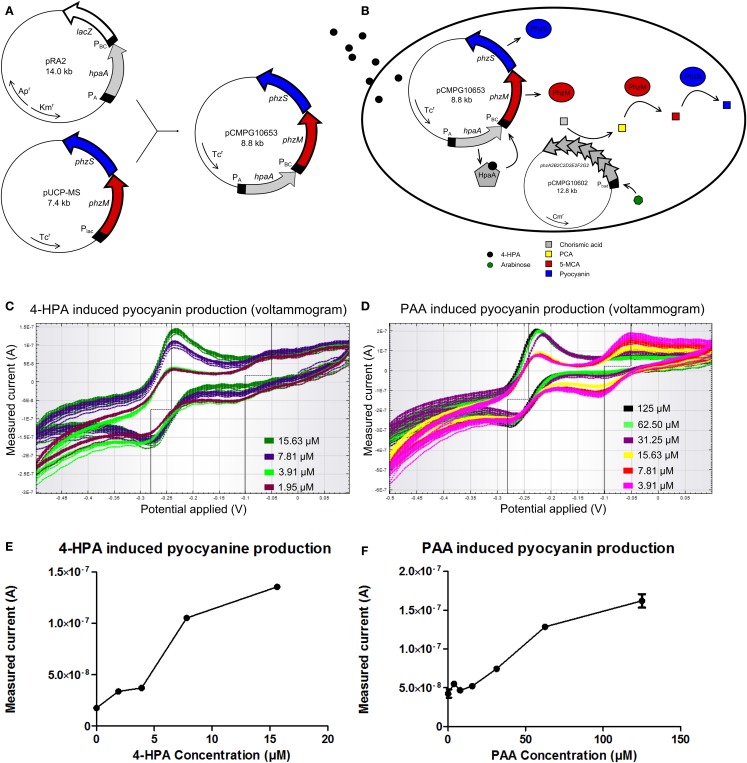
**(A)** Construction of plasmid pCMPG10653. Constitutive promoter P*_A_*, gene *hpaA*, and 4-Hydroxyphenylacetic acid (4-HPA) responsive promoter P*_BC_* were copied from plasmid pRA_2_ (Prieto and García, 1997) and placed in plasmid pUCP-MS (Mavrodi et al., 2001), upstream of gene *phzM*. This yields pyocyanin production controlled by 4-HPA and 2-Phenylacetic Acid (PAA) concentrations, detected by HpaA. **(B)** Schematical overview of the pyocyanin producing bacterial bioassay. In the presence of arabinose, pCMPG10602 expresses proteins to convert chorismic acid to phenazine-1-carboxylic acid (PCA). In the presence of 4-HPA, PhzM and PhzS are expressed. PhzM converts PCA to 5-methylphenazine-1-carboxylicacid (5-MCA), whereas PhzS converts 5-MCA to pyocyanin. **(C)** Superimposition of voltammograms showing the measured current (measure of pyocyanin concentration) in response to 4-HPA, as induced by a cyclic voltage sweep in a CV experiment (4-HPA concentrations green: 15.63 μM, blue: 7.81 μM, light green: 3.91 μM, purple: 1.95 μM). **(D)** Superimposition of voltammograms showing the measured current (measure of pyocyanin concentration) in response to PAA, as induced by a cyclic voltage sweep in a CV experiment (PAA concentrations black: 125 μM, light green: 62.50 μM, purple: 31.25 μM, yellow: 15.625 μM, red: 7.81 μM, pink: 3.91 μM). **(E)** Plot of measured current in function of 4-HPA concentration at an applied voltage of −240 mV. **(F)** Plot of measured current in function of PAA concentration at an applied voltage of −240 mV. *n* = 2 per measured concentration.

A similar rationale as for the GFP producing auxin bioassay was followed for the design of a pyocyanin producing auxin bioassay whose signal can be detected using an electrical readout. For the construction of this bioassay, plasmid pUCP-MS was used as template for the output system. pUCP-MS contains the genes *phzM* and *phzS* (Figure 4A) of *Pseudomonas aeruginosa* PAO1 (Mavrodi et al., 2001). The DNA sequence of pRA_2_ containing the constitutive P*_A_* promoter, the *hpaA* gene, and 4-HPA responsive promoter P*_BC_* was amplified and placed upstream of gene *phzM* in plasmid pUCP-MS to yield pCMPG10653.

Pyocyanin biosynthesis requires precursor molecule PCA. Since PCA is not readily available in *E. coli* TOP10 cells, an additional plasmid was used. Plasmid pCMPG10602 contains the operon *phzA2B2C2D2E2F2G2* from *P. aeruginosa* undercontrol of a P*_BAD_* promoter. The gene products of this operon convert chorismic acid to PCA and their expression is controlled by the presence of arabinose, meaning high doses of PCA will be produced upon induction. Figure 4B shows a schematical overview of the pyocyanin producing bioassay. *E. coli* TOP10 bacteria were transformed with both pCMPG10602, to convert chorismic acid to PCA, and pCMPG10653, to convert PCA to pyocyanin in the presence of auxins. These bacteria were grown in the presence of arabinose and different concentrations of either 4-HPA, PAA, or IAA. Subsequently, the production of pyocyanin in response to the different auxins was measured by means of CV.

During the CV experiment, the induced current is measured in function of the electrical potential that is applied to the culture. The induced current is proportional to the concentration of pyocyanin, since more electrons will be exchanged in the oxidation process of a high concentration of pyocyanin than during oxidation of a low concentration of pyocyanin. Consequently, a high concentration of pyocyanin will induce a higher current than a low concentration of pyocyanin.

Bacteria transformed with pCMPG10653 and pCMPG10602 were exposed to HPA. Measurements of pyocyanin production reveal a dose-response relationship for 4-HPA concentrations from 1.95 to 15.625 μM. This is shown in Figure 4C in the form of a voltammogram where the measured current is shown as a response to the applied voltage, for different concentrations of 4-HPA. Figure 4E shows the measured current in function of 4-HPA concentrations at an applied voltage of −240 mV, the voltage at which pyocyanin oxidizes. Concentrations of 4-HPA above 15.625 μM induced production of pyocyanin but not in a dose-response manner (data not shown). A dose-response relationship can also be seen in the presence of PAA, at concentrations from 15.625 to 125 μM, a broader range than in response to 4-HPA. This is shown in Figure 4D as a voltammogram and Figure 4F as a plot of the measured current in function of PAA concentrations at an applied voltage of −240 mV. Concentrations of PAA above 125 μM induced production of pyocyanin but not in a dose-response manner (data not shown). IAA elicits no measurable production of pyocyanin at any concentration (Figure S10 in Supplementary Material).

In both Figures 4C,D, another notable feature of interest can be seen. Both voltammograms show an additional peak of oxidation at −50 mV and an inverse peak of reduction at −100 mV.

## 3 Discussion

In this work, two whole cell bacterial auxin bioassays were designed and constructed. In both cases, the auxin responsive element HpaA was used as an input module. One incorporated a GFP output module and resulted in a fluorescent bioassay, the second employed a pyocyanin output module, resulting in an electrochemical bioassay.

The GFP producing auxin bioassay (bacteria transformed with pCMPG10652) shows a dose-response relationship in the presence of 4-HPA and PAA concentrations from 60 μM to 3 mM, after an incubation period of 10 h. The pyocyanin producing auxin bioassay (bacteria transformed with pCMPG10602 and pCMPG10653) shows a dose-response to 4-HPA concentrations from 1.9 to 15.625 μM and PAA concentrations from 15.625 to 125 μM. Giridhar et al. demonstrated that externally added PAA concentrations between 3.7 and 15 μM are relevant concentrations for plant growth (Dhaka and Kothari, 2002; Giridhar et al., 2003); other studies report concentrations as high as 100 μM (Ziauddin et al., 1992). Both auxin bioassays are therefore able to detect environmentally relevant concentrations.

The results obtained with both auxin bioassays are comparable to those obtained with other whole cell bacterial bioassays constructed for use in environmental conditions. A large range of required incubation times has been reported for existing bacterial bioassays, ranging from 30 min to 24 h, with the average bacterial bioassay requiring 8–10 h of incubation (Hynninen, 2009; Liu et al., 2010; Behzadian et al., 2011). Detection limits are typically between 0.1and 10 μM (Kragelund et al., 1997; Jaeger et al., 1999; Joyner and Lindow, 2000; Stocker et al., 2003; Behzadian et al., 2011). This corresponds well with the bacterial bioassays that we have constructed in this work.

When comparing both of our auxin bioassays, it can be concluded that the GFP producing auxin bioassay is capable of detecting a broader range of concentrations and higher concentrations of both 4-HPA and PAA, while the pyocyanin producing bioassay is more sensitive to lower concentrations of 4-HPA and PAA. The GFP-producing bioassay requires a fluorescence reader for quantification and may therefore be less easily adapted for use *in situ* than the pyocyanin producing auxin bioassay.

The noted difference in sensitivity between both reporter systems can have multiple causes. Foremost might be the greater complexity of the pyocyanin reporter system. While the GFP output system is built from a single gene, the pyocyanin reporter system consists of two operons. Both operons encode several enzymes that convert intermediates to a single end product. The synthesis of the end product can easily be blocked or delayed in such a long chain of conversions.

Both bioassays are specific to 4-HPA and PAA, since they fail to adequately detect IAA. Figure 1 shows that IAA differs sufficiently in structure from PAA and 4-HPA to explain that HpaA is unable to bind IAA. All other natural auxins have a structure similar to IAA, but further research is necessary to determine the selectivity of both bioassays. High selectivity would allow studying the effects of a combination of auxins, whereby the concentration of 4-HPA or PAA can be measured specifically. On the other hand, this selectivity excludes a number of auxins from detection, reducing the auxin range of the bioassay.

The additional peak of oxidation at −50 mV observed in Figures 4C,D cannot be attributed to the presence of pyocyanin. This indicates that another redox active molecule is being produced in the presence of low amounts of auxins. This molecule is likely also a phenazine or a phenazine precursor since it oxidizes at a negative voltage, but could not be identified yet. There are a number of possible candidates for this phenazine since the conversion of chorismic acid to pyocyanin has many intermediates. A likely candidate is PCA because this intermediate is produced continuously by the products of plasmid pCMPG10602, while, at lower concentrations of auxins, the expression of *phzM* and *phzS*, which convert PCA, is low. This would cause an accumulation of PCA. Nevertheless, the oxidation of PCA happens at −400 mV (Bellin et al., 2014) and not at −50 mV. The only intermediate that oxidizes at approximately −50 mV is 5-MCA, whose oxidation occurs at −70 mV and reduction at −100 mV (Bellin et al., 2014). 5-MCA is thus most likely the intermediate phenazine that is visible on the voltammograms. 5-MCA is converted from PCA by PhzM and is further converted to pyocyanin by PhzS. Accumulation of 5-MCA may suggest that the expression of *phzS* is lower than that of *phzM* at low concentrations of auxins. Further research is necessary to confirm the identity of this accumulating molecule. Additionally, more research is needed to determine the validity of a working *in situ* bioassay, for example, whether the sensors are suitable for use with soil and plant samples. Similarly, engineering of transcription factor HpaA should improve on the sensitivity and selectivity of both bioassays to produce a high quality 4-HPA and PAA bioassay. Complementary research is already being performed for the development of an IAA sensor. This proof of concept shows that the whole cell auxin bioassay is a promising method for auxin detection and can become an important part of auxin research.

## 4 Materials and Methods

### 4.1 Reagents and bacterial cultures

All reagents were purchased from Sigma Aldrich. All auxin solutions were diluted in dimethyl sulfoxide (DMSO). All experiments were performed with *E. coli* TOP10 strains, purchased from Life Technologies Europe. *E. coli* cultures were grown at 37°C in standard Luria Bertani medium in the presence of either 100 μg/mL ampicillin for pCMPG10652 or 25 μg/mL chloramphenicol and 10 μg/mL tetracyclin for pCMPG10602 and pCMPG10653.

### 4.2 Plasmid construction

#### 4.2.1 Construction of pCMPG10602

A *Hin*dIII fragment of pUCP-A2G2 (Mavrodi et al., 2001) containing operon *phzA2B2C2D2E2F2G2* of *P. aeruginosa* was cloned into pBAD33 to construct pCMPG10602 (Table 1). pCMPG10602 was maintained with 25 μg/mL chloramphenicol.

**Table 1:** **Overview of plasmids used in this study**.

Plasmids	Vector	Promoter	Output
pCMPG10602	pBAD33	P*_BAD_*	PhzA2B2C2D2E2F2G2
pCMPG10652	pFPV25	P*_BC_*	GFP
pCMPG10653	pUCP-MS	P*_BC_*	PhzM and PhzS

#### 4.2.2 Construction of pCMPG10652

The DNA sequence containing promoter P*_A_*, *hpaA*, and promoter P*_BC_* of pRA_2_ (Prieto and García, 1997) was amplified using primers PRO6977 AAAAAAGAATTCAGCAGGCGATCGGTATTG and PRO7618 AAAAAATCTAGAATCGGTTGTCCGCCTCTAC. This PCR product was then cloned into pFPV25 (Valdivia and Falkow, 1996) using *Eco*RI and *Xba*I to give pCMPG10652 (Table 1). pCMPG10652 was maintained with 100 μg/mL ampicillin.

#### 4.2.3 Construction of pCMPG10653

The DNA sequence containing promoter P*_A_*, *hpaA*, and promoter P*_BC_* of pRA_2_ (Prieto and García, 1997) was amplified using primers PRO7995 ATGAATTCATTGAGCAGGCGATCGGTAT and PRO8160 ATGAATTCGCTGTTTCCTGTGTGATAAAGAA. This PCR product was then cloned into pUCP-MS (Mavrodi et al., 2001) using *Eco*RI to give pCMPG10653 (Table 1). pCMPG10652 was maintained with 10 μg/mL tetracyclin.

### 4.3 Fluorescence measurements

To determine the influence of bacterial growth phase on GFP production, *E. coli* TOP10 bacteria transformed with pCMPG10652 were grown overnight at 37°C in 5 mL LB medium with 100 μg/mL ampicillin. The following day, these cultures were diluted 1:100 in 200 μL fresh LB medium in fluorescence microtiter plates (Greiner Bio-One) in the presence of 100 μg/mL ampicillin and an auxin solution of a certain concentration, which was added either during lag phase, exponential phase, or stationary phase. Replicates were grown in parallel on the same day. The plates were cultured for 24 h at 37°C and the amount of fluorescence was measured every 2 h using a Synergy MX microtiter plate reader (Biotek Instruments, Inc.).

All further experiments were performed by growing *E. coli* TOP10 bacteria transformed with pCMPG10652 overnight at 37°C in 5 mL LB medium with 100 μg/mL ampicillin. The following day, these cultures were diluted 1:100 in 200 μL fresh LB medium in fluorescence microtiter plates (Greiner Bio-One) in the presence of 100 μg/mL ampicillin and an auxin solution of a certain concentration which was added 3 h after dilution. Replicates were grown in parallel on the same day. The plates were cultured for 10 h at 37°C after which the amount of fluorescence was measured using a Synergy MX microtiter plate reader (Biotek Instruments, Inc.). The fluorescence intensities were normalized with respect to optical density measurements at 595 nm.

### 4.4 Cyclic voltammetry measurements

*E. coli* TOP10 bacteria transformed with pCMPG10602 and pCMPG10653 were grown overnight at 37°C in 5 mL LB medium with 25 μg/mL chloramphenicol and 10 μg/mL tetracyclin. These cultures were diluted 1:100 in 5 mL fresh LB medium in tubes in the presence of an auxin solution of a certain concentration and 25 μg/mL chloramphenicol and 10 μg/mL tetracyclin. The tubes were incubated for 24 h at 37°C, after which a CV experiment was performed. In a CV measurement, the potential of the working electrode is increased linearly over time. When a certain set potential has been reached, the potential is similarly decreased linearly. During one experiment, this can happen multiple times. The current at the working electrode is measured and plotted in function of the applied potential; this results in a cyclic voltammogram (Kissinger and Heineman, 1983).

The CV measurements were performed using a μAutolab type III computer-controlled potentiostat (Metrohm) on a 60EcoMEA-Glass (Multi Channel Systems). The experimental setup was a three-electrode configuration with the MEA as the working electrode, a Red Rod reference electrode (Radiometer Analytical), and a coiled platinum wire as counter electrode. The MEA is connected to the potentiostat through a PCB board, which allows to connect the MEA contact pads with the connection clamps of the potentiostat. During the experiment, the potentiostat cycled from −0.5 V until 0.1 V, 20 times with a 2 mV step potential.

Pyocyanin oxidizes, like all phenazines, at a negative voltage, −247 mV (Bellin et al., 2014; Webster et al., 2014), which is visible as a peak in the current at −240 mV in Figures 4C,D. The reduction of pyocyanin occurs at −300 mV (Bellin et al., 2014), which is visible by the inverse peak at −280 mV. The height of the peak is a measure of the amount of pyocyanin present in the cell, whereby higher amounts of pyocyanin induce a higher current when a voltage is applied (Bellin et al., 2014).

## Author Contributions

JV and WE conceived and supervised the project. SD designed and performed all the experiments, and wrote the manuscript. LV constructed plasmid pCMPG10602. SP revised the manuscript. All authors read and approved the manuscript.

## Conflict of Interest Statement

The authors declare that the research was conducted in the absence of any commercial or financial relationships that could be construed as a potential conflict of interest.

## Supplementary Material

The Supplementary Material for this article can be found online at http://journal.frontiersin.org/article/10.3389/fbioe.2015.00088
