# Supplementary material for: Design and Construction of a Whole Cell Bacterial 4-Hydroxyphenylacetic Acid and 2-Phenylacetic Acid Bioassay
Source: Front Bioeng Biotechnol. 2015 Jun 16;3:88. doi: 10.3389/fbioe.2015.00088 (PMC4468947; doi:10.3389/fbioe.2015.00088)
Supplement: Supplementary file 1 [file presentation_1.pdf]

frontiers

Supplementary Material: Design and construction of a whole cell bacterial 4-hydroxyphenylacetic acid and 2-phenylacetic acid biosensor

Seppe Dierckx [1,*], Sandra Van Puyvelde [1], Lyn Venken [1], Wolfgang Eberle [2] and Jos Vanderleyden [1]

[1] *Centre of Microbial and Plant Genetics, KU Leuven, Leuven , Belgium*
[2] *Imec, Leuven, Belgium*

Correspondence*:
Seppe Dierckx
Centre of Microbial and Plant Genetics, KU Leuven, Kasteelpark Arenberg 20, Leuven, B-3001, Belgium, seppe.dierckx@biw.kuleuven.be

1 SUPPLEMENTARY FIGURES

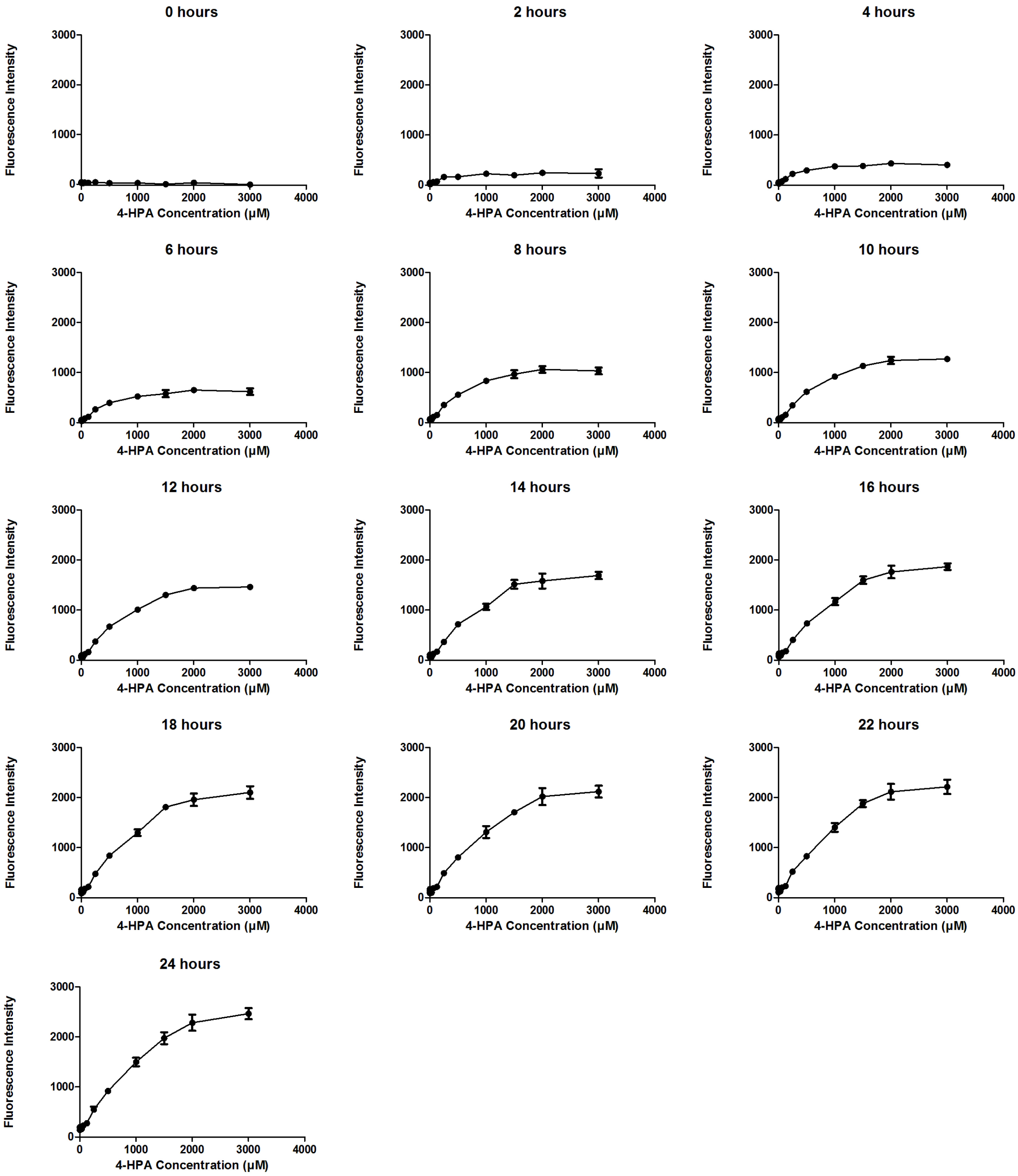

Supplementary Figure 1. Fluorescence measurements of GFP production of strain pCMPG10652/ *E. coli* TOP10 in response to a range of 4-hydroxyphenylacetic acid concentrations, induced during exponential phase. Measurements were taken every 2 hours for 24 hours. Auxin concentrations used in fluorescence measurements: 3 mM, 2mM, 1.5mM, 1 mM, 500 μM, 250 μM, 125 μM, 62 μM, 31.25 μM, 15.625 μM and 0 M. n = 8 per measured concentration.

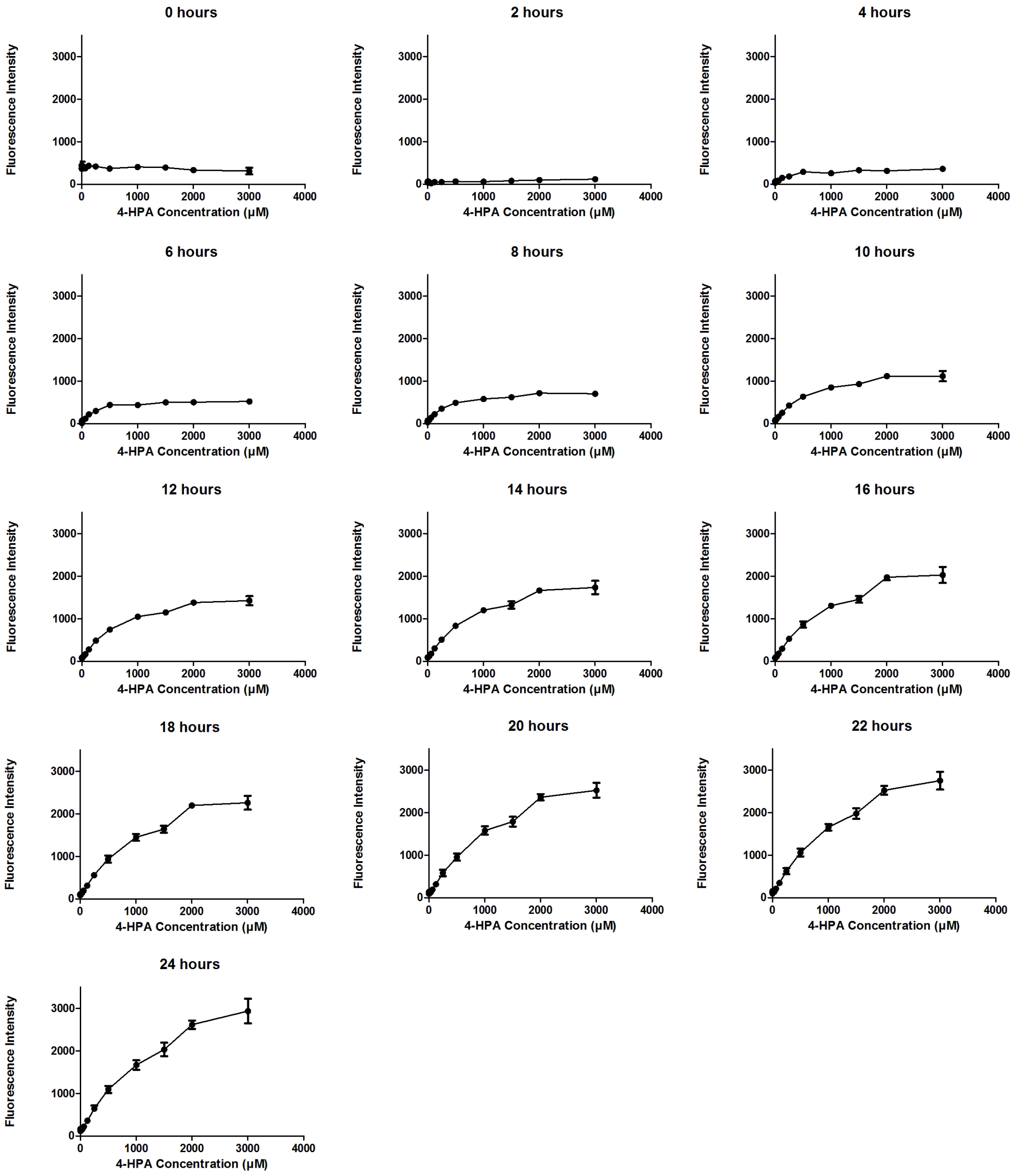

Supplementary Figure 2. Fluorescence measurements of GFP production of strain pCMPG10652/ *E. coli* TOP10 in response to a range of 4-hydroxyphenylacetic acid concentrations, induced during lag phase. Measurements were taken every 2 hours for 24 hours. Auxin concentrations used in fluorescence measurements: 3 mM, 2mM, 1.5mM, 1 mM, 500 μM, 250 μM, 125 μM, 62 μM, 31.25 μM, 15.625 μM and 0 M. n = 8 per measured concentration.

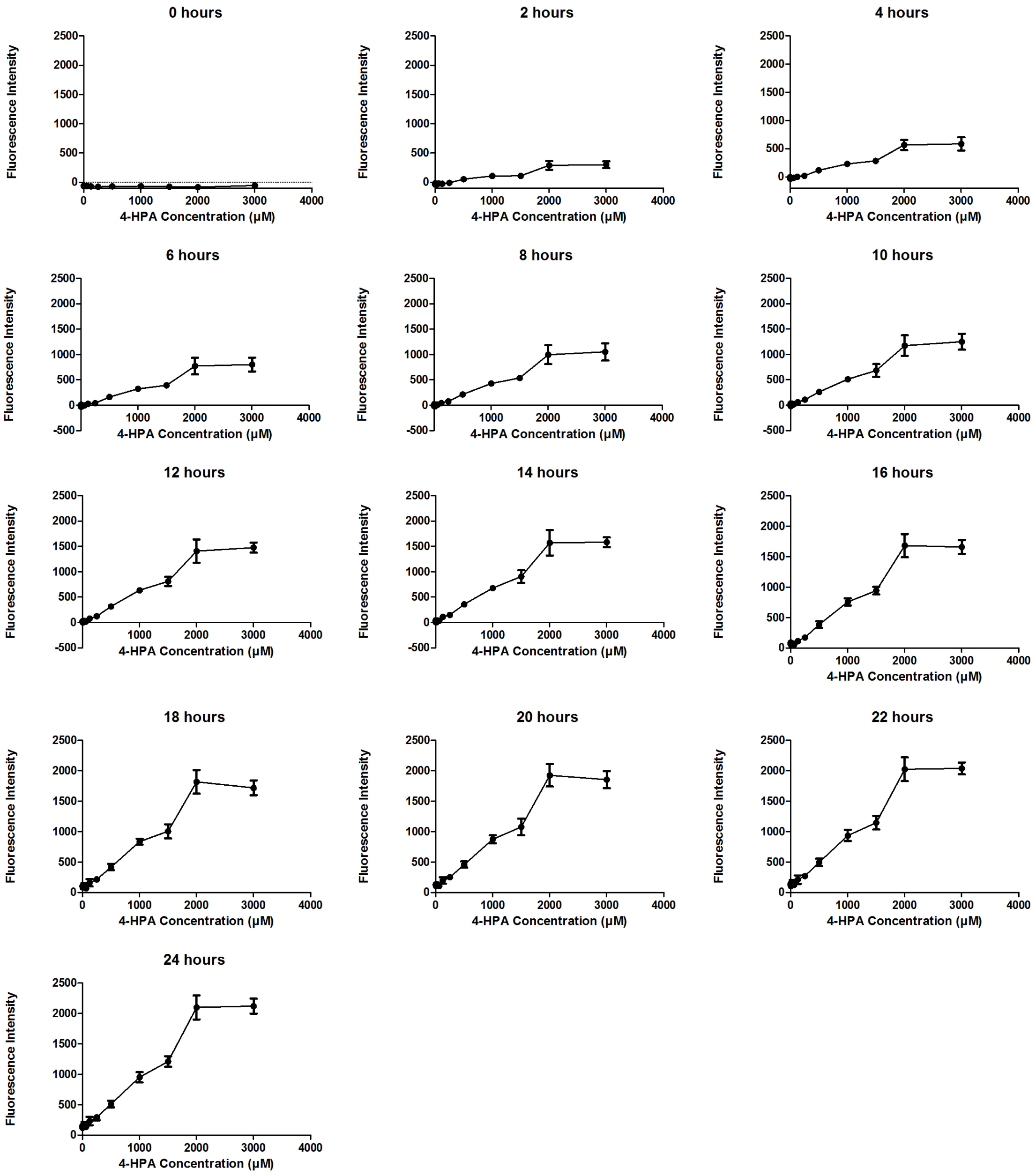

Supplementary Figure 3. Fluorescence measurements of GFP production of strain pCMPG10652/ *E. coli* TOP10 in response to a range of 4-hydroxyphenylacetic acid concentrations, induced during stationary phase. Measurements were taken every 2 hours for 24 hours. Auxin concentrations used in fluorescence measurements: 3 mM, 2mM, 1.5mM, 1 mM, 500 μM, 250 μM, 125 μM, 62 μM, 31.25 μM, 15.625 μM and 0 M. n = 8 per measured concentration.

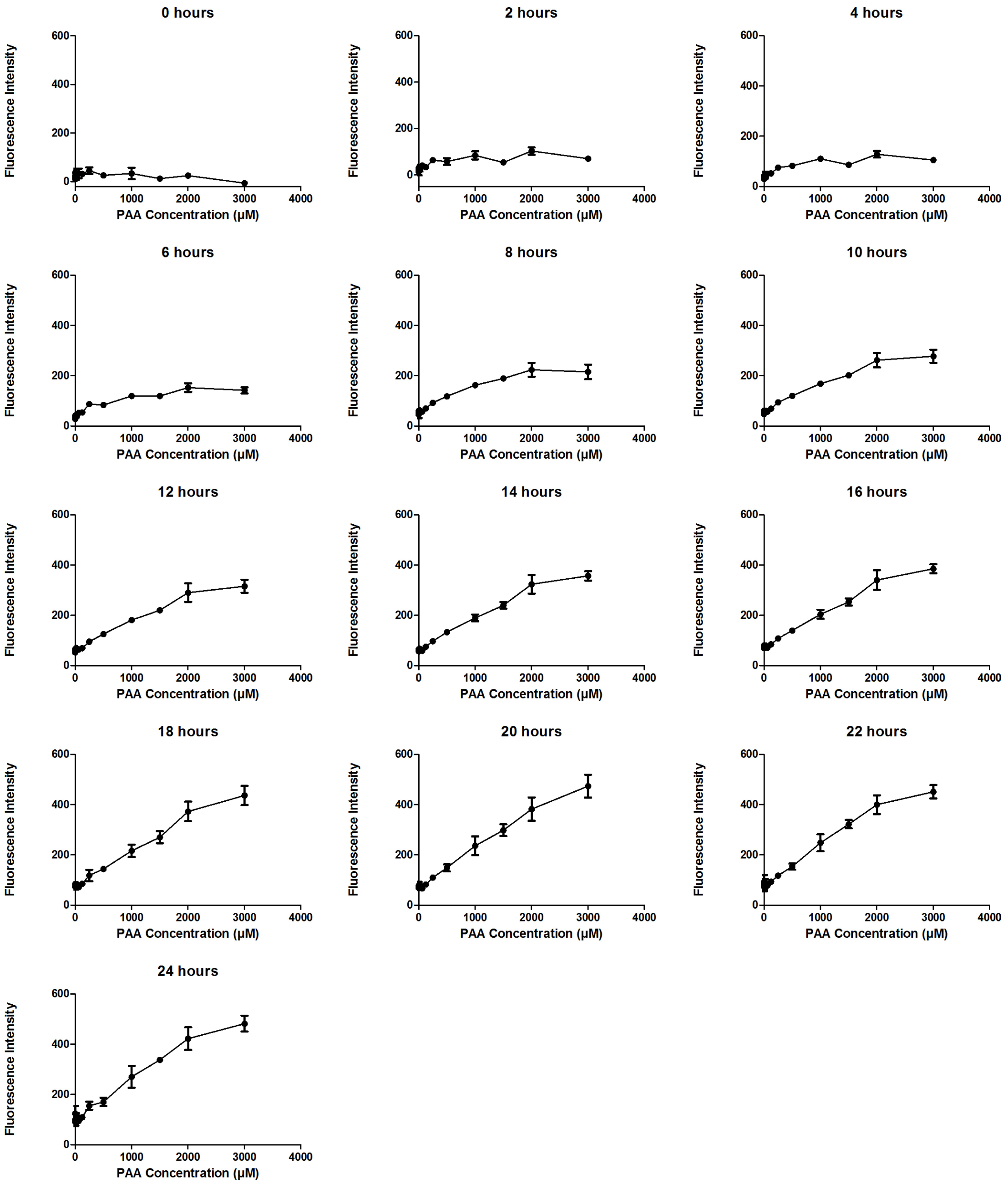

Supplementary Figure 4. Fluorescence measurements of GFP production of strain pCMPG10652/ *E. coli* TOP10 in response to a range of 2-phenylacetic acid acid concentrations, induced during exponential phase. Measurements were taken every 2 hours for 24 hours. Auxin concentrations used in fluorescence measurements: 3 mM, 2mM, 1.5mM, 1 mM, 500 μM, 250 μM, 125 μM, 62 μM, 31.25 μM, 15.625 μM and 0 M. n = 8 per measured concentration.

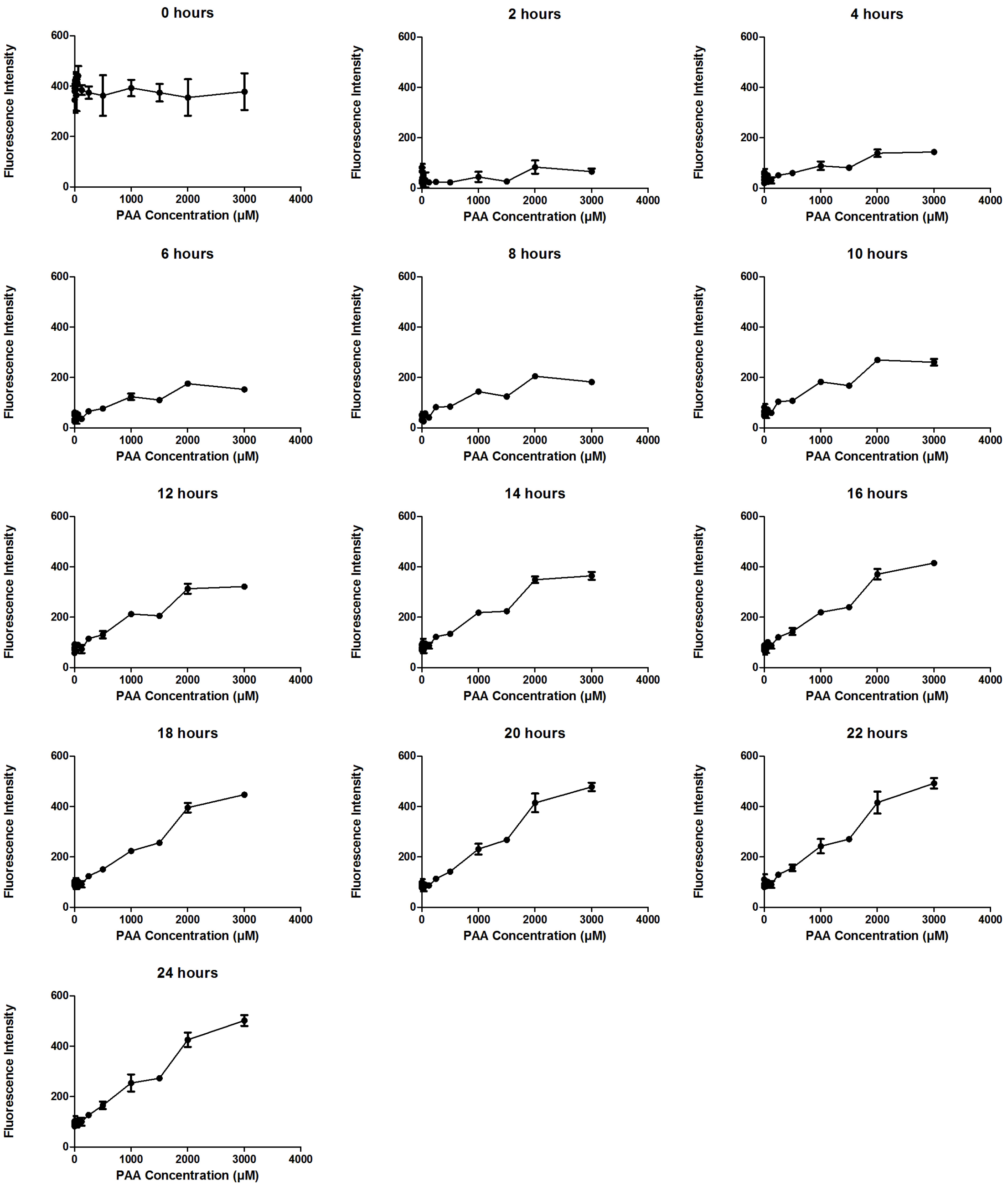

Supplementary Figure 5. Fluorescence measurements of GFP production of strain pCMPG10652/ *E. coli* TOP10 in response to a range of 2-phenylacetic acid acid concentrations, induced during lag phase. Measurements were taken every 2 hours for 24 hours. Auxin concentrations used in fluorescence measurements: 3 mM, 2mM, 1.5mM, 1 mM, 500 μM, 250 μM, 125 μM, 62 μM, 31.25 μM, 15.625 μM and 0 M. n = 8 per measured concentration.

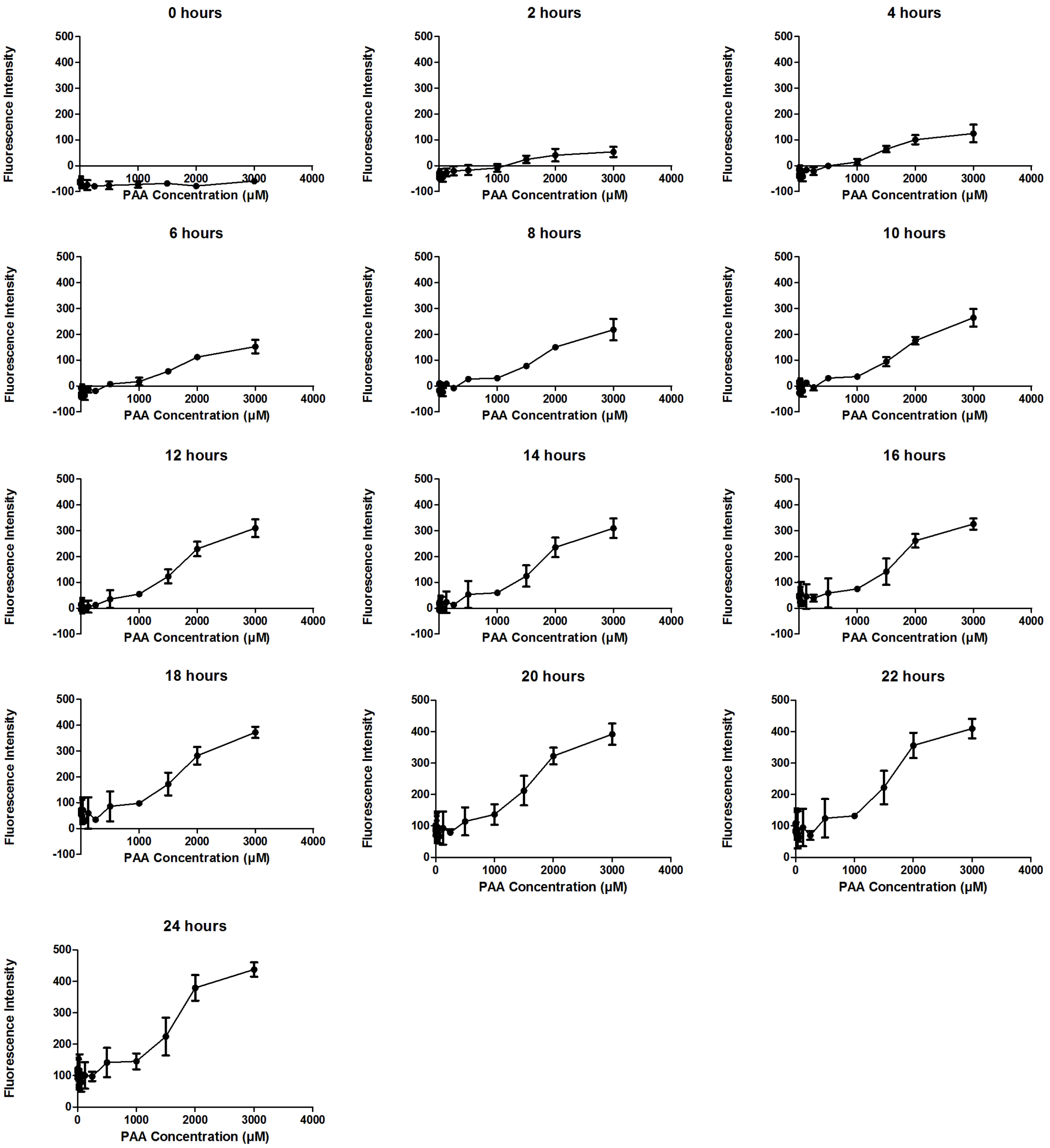

Supplementary Figure 6. Fluorescence measurements of GFP production of strain pCMPG10652/ *E. coli* TOP10 in response to a range of 2-phenylacetic acid acid concentrations, induced during stationary phase. Measurements were taken every 2 hours for 24 hours. Auxin concentrations used in fluorescence measurements: 3 mM, 2mM, 1.5mM, 1 mM, 500 μM, 250 μM, 125 μM, 62 μM, 31.25 μM, 15.625 μM and 0 M. n = 8 per measured concentration.

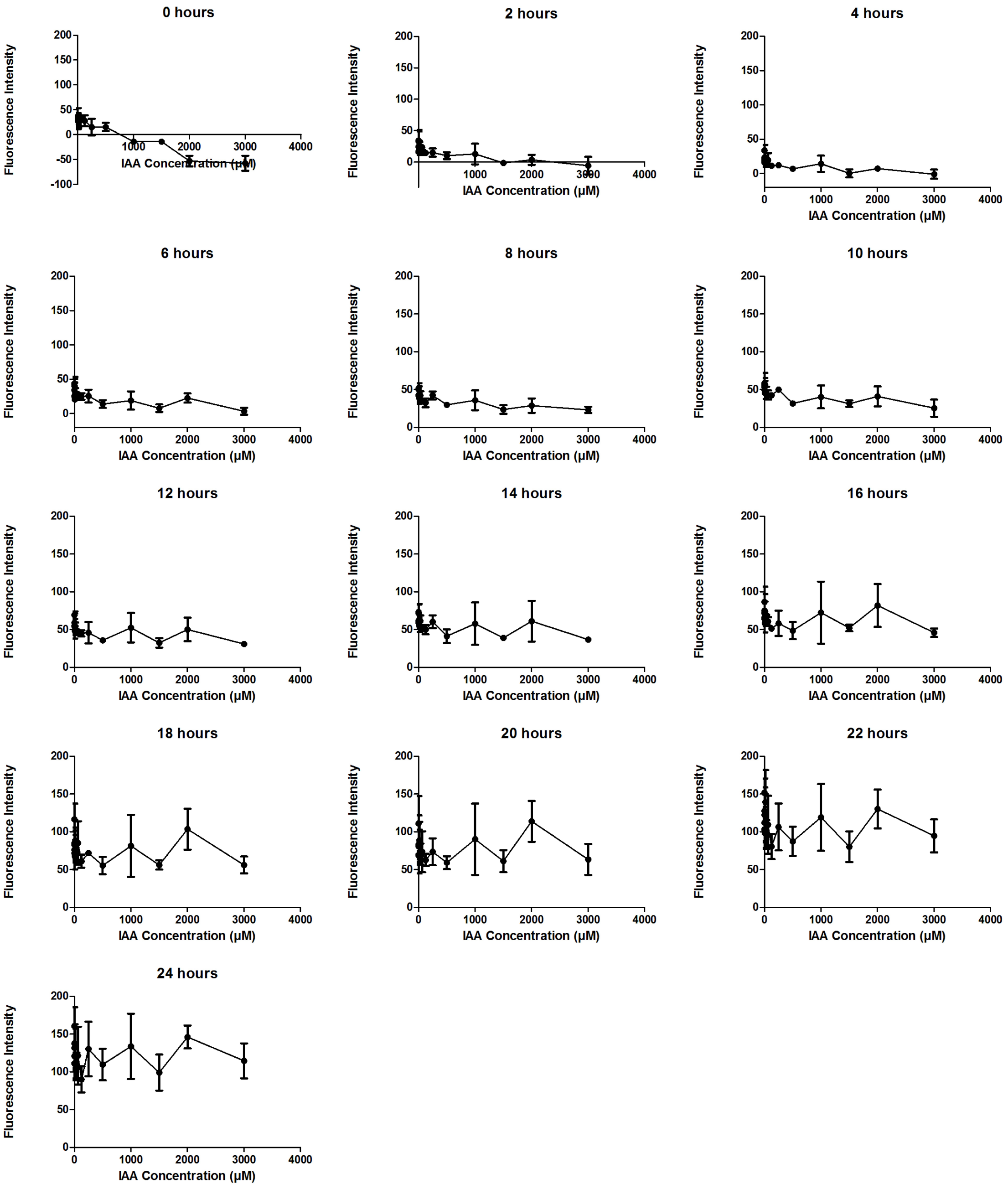

Supplementary Figure 7. Fluorescence measurements of GFP production of strain pCMPG10652/ *E. coli* TOP10 in response to a range of indole-3-acetic acid concentrations, induced during exponential phase. Measurements were taken every 2 hours for 24 hours. Auxin concentrations used in fluorescence measurements: 3 mM, 2mM, 1.5mM, 1 mM, 500 μM, 250 μM, 125 μM, 62 μM, 31.25 μM, 15.625 μM and 0 M. n = 8 per measured concentration.

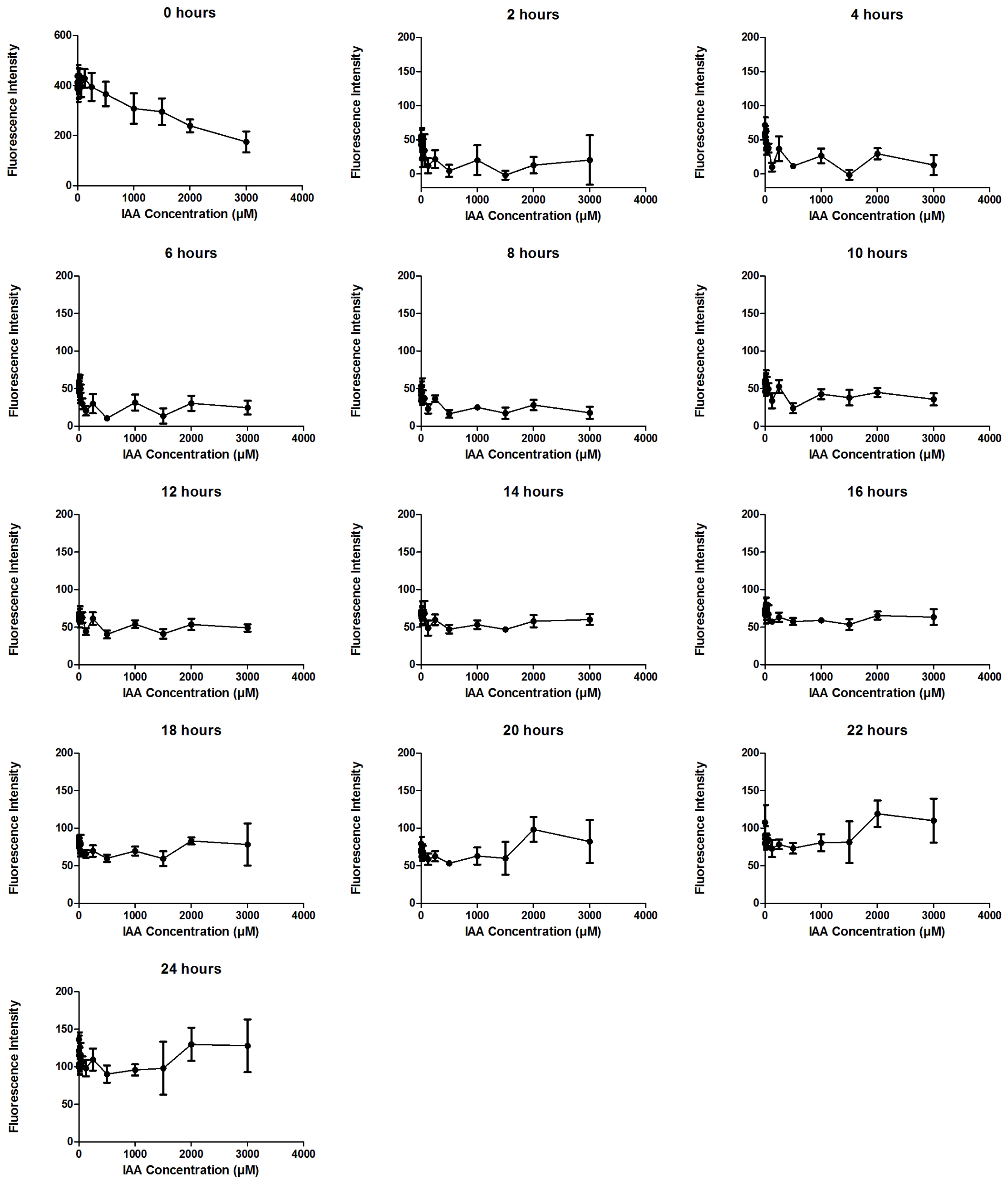

Supplementary Figure 8. Fluorescence measurements of GFP production of strain pCMPG10652/ *E. coli* TOP10 in response to a range of indole-3-acetic acid concentrations, induced during lagl phase. Measurements were taken every 2 hours for 24 hours. Auxin concentrations used in fluorescence measurements: 3 mM, 2mM, 1.5mM, 1 mM, 500 μM, 250 μM, 125 μM, 62 μM, 31.25 μM, 15.625 μM and 0 M. n = 8 per measured concentration.

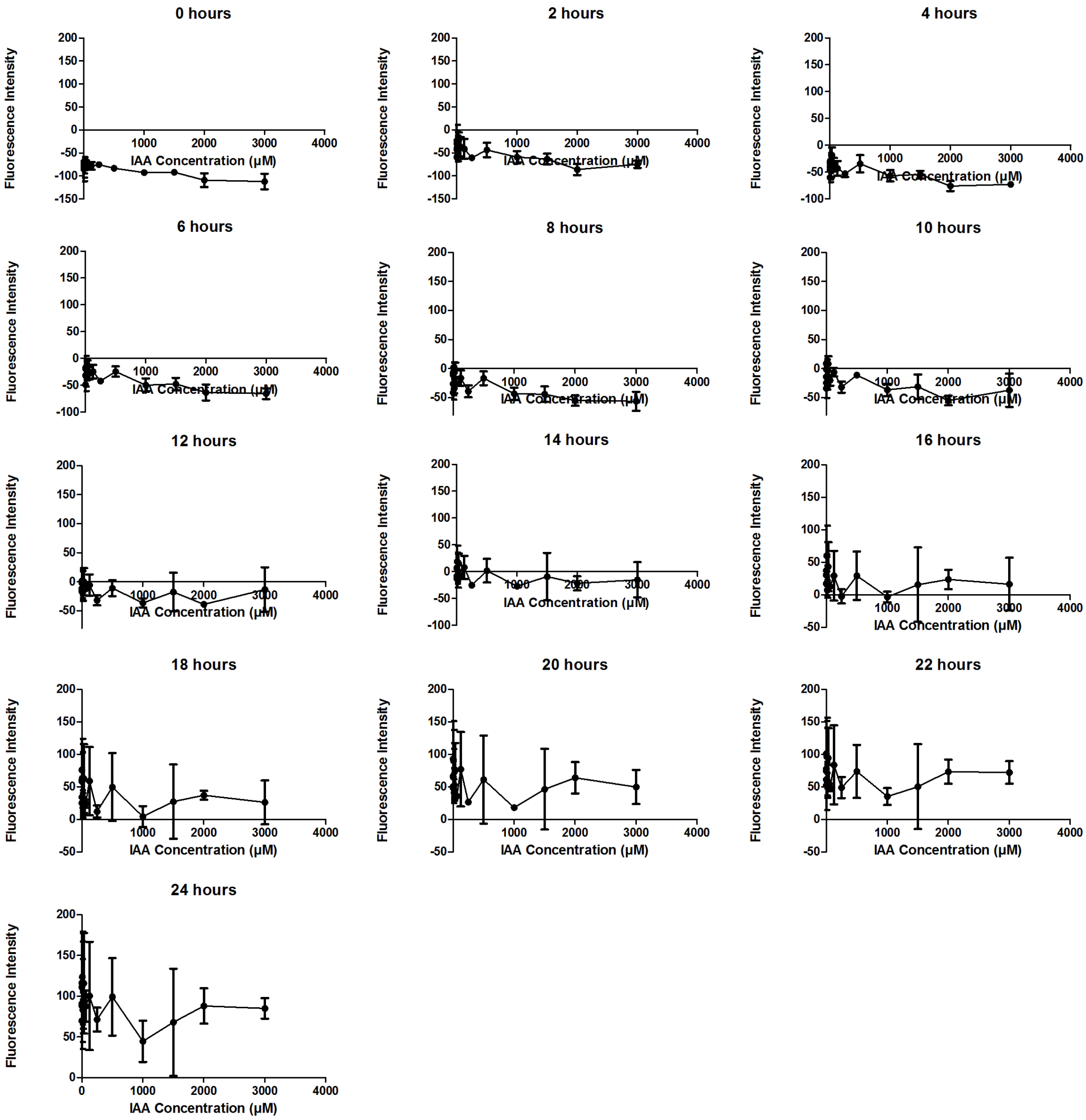

Supplementary Figure 9. Fluorescence measurements of GFP production of strain pCMPG10652/ *E. coli* TOP10 in response to a range of indole-3-acetic acid concentrations, induced during stationary phase. Measurements were taken every 2 hours for 24 hours. Auxin concentrations used in fluorescence measurements: 3 mM, 2mM, 1.5mM, 1 mM, 500 μM, 250 μM, 125 μM, 62 μM, 31.25 μM, 15.625 μM and 0 M. n = 8 per measured concentration.

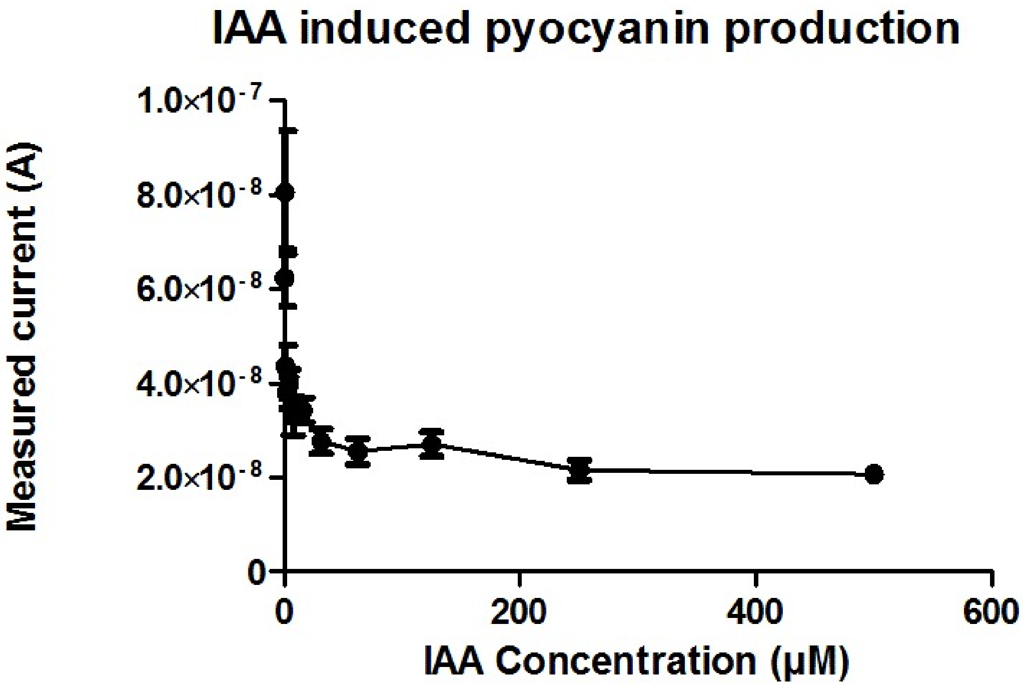

Supplementary Figure 10. Plot of measured current in function of indole-3-acetic acid concentration at an applied voltage of -240 mV. n = 2 per measured concentration.
